# Costs and Benefits of Experimentally Induced Changes in the Allocation of Growth versus Immune Function under Differential Exposure to Ectoparasites

**DOI:** 10.1371/journal.pone.0010814

**Published:** 2010-05-25

**Authors:** Natalia Pitala, Heli Siitari, Lars Gustafsson, Jon E. Brommer

**Affiliations:** 1 Bird Ecology Unit, Department of Biological and Environmental Sciences, University of Helsinki, Helsinki, Finland; 2 Department of Biological and Environmental Science, University of Jyväskylä, Jyväskylä, Finland; 3 Department of Animal Ecology/Ecology and Evolution, Evolutionary Biology Centre, Uppsala University, Uppsala, Sweden; University of Oxford, United Kingdom

## Abstract

**Background:**

Ecological immunology has focused on the costs of investment in immunocompetence. However, understanding optimal resource allocation to immune defence requires also identification of its benefits, which are likely to occur only when parasites are abundant.

**Methodology:**

We manipulated the abundance of parasitic hen fleas in blue tit (*Cyanistes caeruleus*) nests, and supplemented their hosts, the nestlings, with methionine (a sulphur amino acid enhancing cell-mediated immunity) during day 3–6. We found a significant interaction between these two experimental factors on the development of immune defences and growth rates. Only in parasitized nests did methionine supplementation boost immune (PHA) response, and did nestling with experimentally increased immunocompetence show a relatively faster growth rate than control nestlings between days 6–9. Hence, the allocation of resources into immune defence and its growth-benefits are apparent only in presence of parasites. The main cost of methionine-induced increased allocation to the immune system was an increase in mortality, independently of ectoparasites. Nestlings in all treatments compensated initial growth reduction and all reached equal body size at day 16 (just prior to fledging), indicating a lack of long-term benefits. In addition, methionine treatment tended (P = 0.09) to lower circulating plasma immunoglobulin levels, possibly indicating a trade-off between the cell-mediated and humoral components of the immune system.

**Conclusions:**

We found no strong benefits of an increased investment in immunocompetence in a parasite-rich environment. Any deviation from the growth trajectory (due to changes in allocation induced by methionine) is largely detrimental for survival. Hence, while costs are apparent identifying the benefits of investment in immunocompetence during ontogeny is challenging.

## Introduction

In order to understand the significance of immune defence and its role in life histories, it is essential to assess its associated costs and benefits. Evolutionary ecology has focused on the costs of immune defence [1]. These costs can be attributed to building up the immune machinery and maintaining a competent immune system, and to using it, i.e. responding to an immunological challenge [2], [3]. Investments in immune defences can be effectively manipulated during ontogeny. Direct manipulation of immunity in order to tear apart its relationship with condition is a particularly powerful approach to demonstrate the causal relationship between a fitness component (e.g. growth, survival) and immunity [4], [5]. Four studies to date have carried out such a manipulation in wild bird populations by supplementing nestlings with methionine [6], [7], [8], [9]. Methionine is a sulphur amino-acid, essential in diet. It acts as an antioxidant and takes part in metabolism of lipids. Methionine shows immunoenhancing properties. It is involved in synthesis of glutathione [10], of which intracellular concentration affects functions of T-cells, such as cytotoxic properties and proliferation [10,11,1213,14]. Oral administration of sulphur amino acids may also directly influence T-cell production [15].

In birds, methionine supplementation causes elevated *in vivo* response to subcutaneously injected phytohaemagglutinin (PHA) [e.g. 6], [7], [16], a composite measures of cell-mediated immunocompetence [17]. Enrichment of natural nestlings' diet in methionine, applied in wild bird studies, does not change the amount of resources (proteins or energy) available to nestlings for growth or development of physiological functions, but should alter the allocation of available resources [6] by stimulating nestlings to increase their investment in immune system. Using methionine supplementation, it has been shown that a reduction in growth is a cost associated with increased investment in immunity [6], [7].

The benefits of investing in immune defence have received far less attention than the costs, probably because these benefits seem, at first hand, to be obvious. An improved immune defence against diseases is thought to increase lifespan and, in consequence, fitness [2]. Nonetheless, the available evidence linking survival (lifespan) to immunocompetence [18], [19], [20], [21] is correlative. This correlation is clearly undermined by condition-dependence of both survival and immunocompetence [5] − a higher amount of resources at an individual's disposal is likely to increase immune defence as well as survival, irrespective of whether there is a causal link between them. The most obvious immediate benefit of enhanced immune function is an improved protection against parasites and pathogens. PHA response measures an effective immunoreaction against ectoparasites: Ectoparasites take smaller bloodmeals from nestlings that had their immune system boosted with methionine [9], and a high nestling PHA response reduces the fecundity of ectoparasites [22]. In order to study both costs and benefits of immunocompetence, we here stimulate the immune system of blue tit *Cyanistes caeruleus* nestlings in the absence and presence of a common nest ectoparasite, the hen flea *Ceratophyllus gallinae*. Ectoparasites are harmful to nestlings, both because they drain blood (reduce nutrients), and because they may transfer pathogens into the host. We expect that nestlings with an experimentally increased immunocompetence reap benefits (relative to control nestlings) in the presence of parasites, whereas costs should dominate in absence of parasites. In order to get a more complete picture of the effects of methionine on the components of the immune system, we measured, in addition to PHA-response, the level of circulating immunoglobulins, as an index of humoral immune function. Although interpretation of general Ig level (measured without antigenic stimulation) in terms of immunocompetence is difficult, it can be regarded as a reflection of general efficiency of B-lymphocytes [23], as in our study nestlings were of the same age and we controlled, to some extent, their environment.

## Results

Our parasite treatment was successful in creating differential exposure of nestlings to hen fleas. Deparasitized nests at the end of the nestling period had 10.4±2.8 (mean ± SE) fleas, whereas parasitized nests had 84.0±13.3 fleas (Mann-Whitney test: U = 27.0, N = 40, p<0.0001). Maximal number of fleas found in a parasitized nest (260) was within the range found in unmanipulated nests (4–280), and the mean number of fleas did not differ between unmanipulated and parasitized nests (U = 201.0, N = 42, p = 0.63). Hence, our experimental infestation resembles the natural level of flea parasitism. Before the start of methionine supplementation nestlings did not differ in body mass between treatments (linear mixed model; methionine: F_1, 388_ = 0.04, p = 0.85; parasites: F_1, 40_ = 0.10, p = 0.75; parasites × methionine: F_1, 388_ = 0.01, p = 0.91).

**Figure 1:**
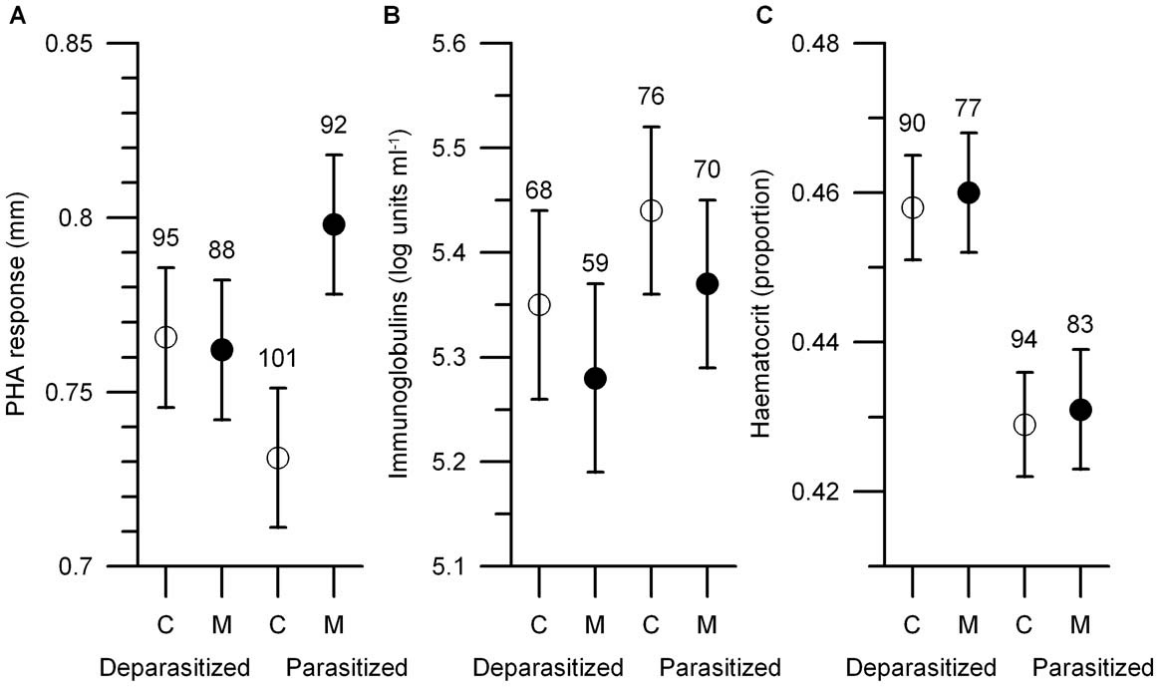
Physiological traits of control (C, open symbols) and methionine-supplemented (M, filled symbols) blue tit nestlings reared in deparasitized and parasitized nests; least square means ± SE. Sample sizes (number of nestlings) are indicated on the graphs.

The effect of methionine treatment on PHA-response depended on ectoparasite abundance (Table 1). Methionine-supplemented nestlings mounted higher immune response to PHA than control nestlings in parasitized nests, whereas PHA-response of nestlings from deparasitized nests did not differ significantly (Table 1, Fig. 1a). There was a tendency of methionine supplementation to decrease nestlings' total Ig levels (Table 1, Fig. 1b), independently of parasite manipulation. PHA-response and total Ig level were not correlated (Pearson's r = −0.04; p = 0.49; N = 272). Haematocrit value was not affected by methionine treatment (Table 1, Fig. 1c), but was clearly lowered by high ectoparasite load (Table 1, Fig. 1c), probably reflecting anaemia caused by the feeding of ectoparasites on their hosts.

**Table 1:** Analysis of the effect of ectoparasite load manipulation (parasites), methionine supplementation (methionine) and interaction between these treatments on physiological traits of blue tit nestlings.

Factor	PHA-response N = 376			Ig level N = 273			Haematocrit N = 344		
	df	F	p	df	F	p	df	F	p
parasites	1, 39	0.00	1.00	1, 34	0.76	0.39	1, 39	9.69	0.004
methionine	1, 333	4.26	0.04	1, 235	2.93	0.09	1, 301	0.17	0.67
interaction	1, 333	5.42	0.02	1, 235	0.05	0.83	1, 301	0.001	0.97

	V	SE	V (%)	V	SE	V (%)	V	SE	V (%)
Brood (parasites)	6.7×10^−3^	1.8×10^−3^	50.0	0.10	0.03	54.6	6.6×10^−4^	2.3×10^−4^	24.5

Statistics of a linear mixed model with brood nested in parasite treatment fitted as a random effect; variance component, its standard error and percentage of variance explained by brood effect nested in the parasite treatment is shown. N equals the number of nestlings assayed.

**Figure 2:**
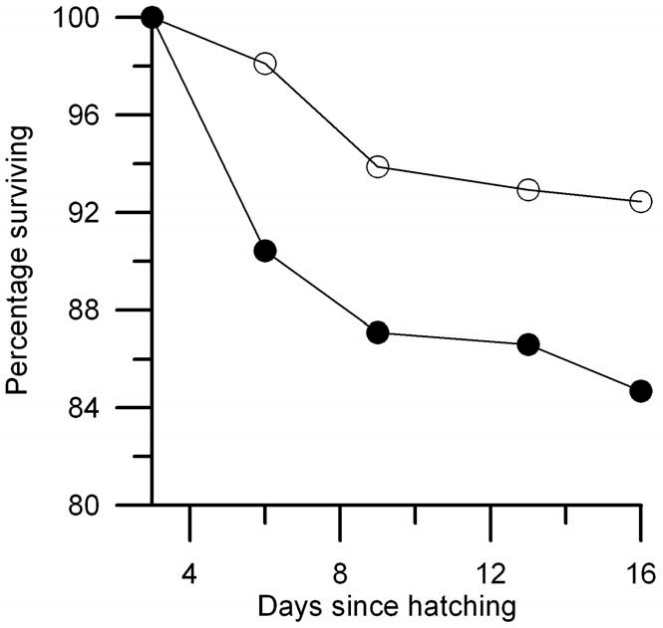
Survival of blue tit nestlings after the start of methionine supplementation (day 3 post-hatching). During the supplementation period (between day 3 and 6) methionine-treated nestlings (M, filled symbols, N = 209) had higher mortality than control nestlings (C, open symbols, N = 212).

Supplementing nestlings with methionine increased their mortality, similarly in deparasitized and parasitized nests (GLMM; methionine treatment: F_1, 376_ = 5.91, p = 0.016; parasite treatment: F_1, 39_ = 0.01, p = 0.92; parasites × methionine: F_1, 376_ = 1.32, p = 0.25; initial body mass: F_1, 376_ = 79.69, p<0.0001, coefficient: −2.55±0.28 (SE); brood: variance = 2.54, 95% CI = (1.23, 5.26)). In the control group the highest mortality occurred between day 6 and 9; after day 9 mortality remained negligible throughout the rest of the nestling period (Fig. 2). In the methionine-treated group, nestling survival was the lowest between day 3 and 6, i.e. during the period of methionine supplementation; after day 6 mortality of supplemented and control nestlings was similar (Fig. 2). Brood size on day 9 did not differ between parasite treatment groups (t_40_ = 0.583, p = 0.563).

**Figure 3:**
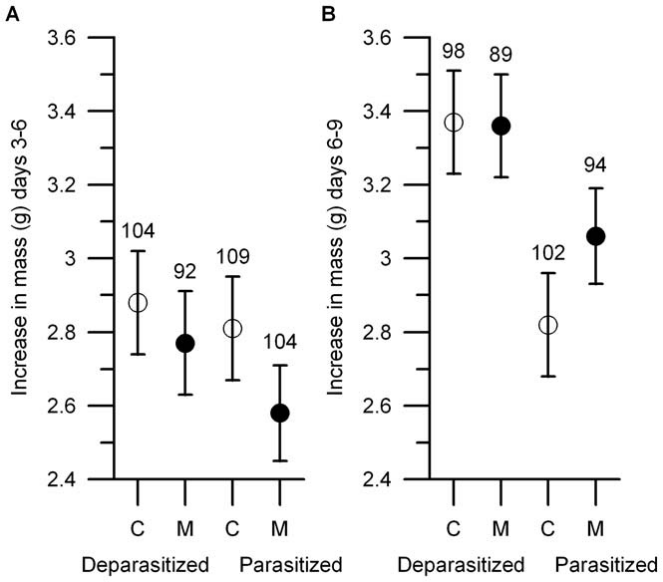
Mass gain of control (C, open symbols) and methionine-supplemented (M, filled symbols) blue tit nestlings in deparasitized and parasitized nests; least square means + SE. (A) During the supplementation period (between day 3 and 6) methionine treated nestlings had suppressed growth, but in deparasitized nests not significantly so. (B) Immediately after supplementation ended (between day 6 and 9) methionine treated nestlings had higher mass gain than control nestlings in parasitized nests, but not in deparasitized nests. Sample sizes are indicated on the graphs. Statistics in Table 2.

During the supplementation period (days 3–6) methionine also suppressed growth of surviving nestlings (Table 2, Fig. 3a). During the short period after the termination of supplementation (days 6–9) methionine-treated nestlings showed increased growth compared to control nestlings (Table 2). This effect depended on parasite loads in nests, as shown by significant interaction between methionine and parasite treatment (Table 2, Fig. 3b). In deparasitized nests, control and methionine-supplemented nestlings did not differ in mass gain (Tukey post-hoc test, p>0.05), but in parasitized nests supplemented nestlings grew faster than control nestlings (Tukey post-hoc test, p<0.05). On day 9, nestlings' body mass was significantly different between deparasitized and parasitized nests (Table 2) but the effect of methionine treatment and its interaction with parasite treatment were no longer detectable (Table 2). Similarly, shortly before fledging (on day 16) methionine supplemented and control nestlings did not differ in any of morphological traits we measured (Table 3). Also the parasite treatment did not affect significantly the final morphology of nestlings (Table 3).

**Table 2:** Analysis of the effect of ectoparasite load manipulation (parasites), methionine supplementation (methionine) and interaction between these treatments on growth of blue tit nestlings during the period of methionine supplementation (days 3–6) and immediately after termination of supplementation (days 6–9, day 9).

factor	body mass gain, days 3–6 N = 409			body mass gain, days 6–9 N = 383			body mass on day 9 N = 383		
	df	F	p	df	F	p	df	F	p
parasites	1, 40	0.85	0.36	1, 40	5.21	0.03	1, 40	5.30	0.02
methionine	1, 365	8.24	0.004	1, 339	4.51	0.03	1, 339	0.10	0.73
interaction	1, 365	0.83	0.36	1,339	5.12	0.02	1, 339	0.46	0.46

	V	SE	V (%)	V	SE	V (%)	V	SE	V (%)
brood (parasites)	0.17	0.50	33.2	0.33	0.09	55.1	0.57	0.16	40.1

Statistics of a linear mixed model with brood nested in parasite treatment fitted as a random effect; variance component, its standard error and percentage of variance explained by brood effect nested in the parasite treatment is shown. N equals the number of nestlings measured.

**Table 3:** Analysis of the effect of ectoparasite load manipulation (parasites) methionine supplementation (methionine) and interaction between these treatments on final body size of blue tit nestlings.

factor	body mass N = 373			tarsus length N = 373			wing length N = 376		
	df	F	p	df	F	p	df	F	p
parasites	1, 39	2.18	0.15	1, 39	0.15	0.70	1, 38	0.56	0.50
methionine	1, 329	0.09	0.76	1, 330	0.01	0.94	1, 325	0.26	0.61
interaction	1, 329	1.28	0.26	1, 330	0.10	0.76	1, 325	0.57	0.45

	V	SE	V (%)	V	SE	V (%)	V	SE	V (%)
brood (parasites)	0.35	0.10	50.9	0.15	0.04	30.5	5.4	1.5	45.9

Statistics of a linear mixed model with brood nested in parasite treatment fitted as a random effect; variance component, its standard error and percentage of variance explained by brood effect nested in the parasite treatment is shown. N equals the number of nestlings measured.

The analysis of recruitment did not indicate that our experimental treatments influenced nestlings' post-fledging performance (GLMM; methionine treatment: F_1, 330_ = 0.68, p = 0.41; parasite treatment: F_1, 39_ = 0.90, p = 0.35; parasites × methionine: F_1, 330_ = 1.19, p = 0.28; brood: variance = 0.979, 95% CI = (0.842, 1.139)). However, recruitment of nestlings was generally low and only 10 out of 373 nestlings that survived until day 16 were found breeding in the study area in the following years.

## Discussion

### Context dependent investment in immune function

The allocation of resources towards cell-mediated immune system of birds can be experimentally increased by supplementary feeding them methionine during development [6], [16]. We here used this method to study the consequences of altered allocation of resources to growth versus immunocompetence, contrasting a deparasitised against a parasite-rich environment. We indeed found a statistically significant main effect of methionine supplementation on PHA response measured seven days after the supplementation ended, but closer scrutiny revealed that methionine supplementation caused a relatively long-term elevation in the PHA-response of nestlings in parasitized nests only (i.e., there was a significant interaction with parasite manipulation). To our knowledge, this is the first evidence that physiological manipulation of immune function interacts with the parasite-environment an individual faces. This finding was not driven by differential mortality where individuals with an exaggerated immune response were selected away in the parasite free environment, because we did not find an interaction between methionine supplementation and hen flea manipulation on mortality. Developing blue tits apparently have a higher propensity to invest in their immune system when facing attacks by parasites. We see two interpretations of this finding.

Firstly, most studies that have shown an effect of methionine on PHA response have been carried out in an environment where ectoparasites were not reduced [6], [7], [9]. Typically, the PHA response is measured some days (in this study, seven days) after methionine supplementation had stopped and thus describes a relatively long-lasting ontogenetic change in immune defences. The clear reduction in growth rates during methionine supplementation indicates that resources are indeed allocated away from growth in both de-parasitised and parasite-rich nests. Based on previous studies [6], [7], [9], it seems plausible that these resources were allocated into immune defence during the period of methionine supplementation. However, hosts that find themselves in a parasite-poor environment may, after the experimental upregulation of allocation to immune defences, simply downregulate this allocation back to regular levels. PHA response is effective against ectoparasites [8], [9] and in a parasite-rich environment the investment in immunity is presumably stimulated. In order to evaluate this interpretation, future work should measure PHA response both directly after the experimental feeding of methionine and when nestlings are fully developed.

Secondly, arthropod ectoparasites have components in their saliva that downregulate the host's immune defences [24]. The physiological pathway involved in this modulation is likely to interact with methionine. Immunomodulatory effects of ectoparasites often involve a shift from T helper 1 (cell-mediated) immune responses to T helper 2 (antibody) responses [24], whereas glutathione (the concentration of which is raised by methionine supplementation) acts in the opposite direction [25], [26]. These physiological processes suggest that methionine supplementation in the presence of hen fleas would cause a much stronger effects than when parasites are absent. In poultry studies (without food stress and without ectoparasites), supplementary feeding of methionine does not always lead to higher immunocompetence, particularly so in low-disease environments [27], [28]. Clearly, more studies that jointly manipulate the parasite environment and immunocompetence under ecologically relevant levels of resource availability are needed in order to address these issues further.

### Costs and benefits of immunocompetence

An experimentally increased immunocompetence should be especially beneficial when there are parasites to fight. Indeed, during three days after methionine treatment had stopped, methionine-supplemented nestlings in parasitized nests grew faster than control nestlings. The same two-factorial design (methionine supplementation and parasite manipulation) as we applied here was used in great tit (*Parus major*) nestlings [8]. The same difference in growth rates was found in this experiment, which was interpreted as evidence that increased investment in immune defence is beneficial when parasite load is high. Nevertheless, we feel that interpreting methionine-modified growth curves of nestlings in parasitized nests as purely beneficial is problematic for two reasons. First, our experimental design controls for variation in hatching date and brood size, which are strong determinants of future recruitment of offspring. Thus, the main fitness benefits in our experiment are likely to be associated with a higher propensity for heavier fledglings to recruit [29], [30], [31], [32] and not with an altered growth rate *per se*. Therefore, ideally, a beneficial artificially enhanced immune defence would result in a higher body mass at fledgling if there are parasites. No such difference in final size has been found in our study. Ref.6 did not analyse the final body size or mass of nestlings, but from the growth rates they present, it can be inferred that both methionine-treated and control nestlings reached similar body mass at fledging. Second, compensatory or catch-up growth, as shown by methionine-supplemented nestlings is actually thought to be costly [reviewed in 33]. Costs of compensatory growth are often expressed much later in life, e.g. as a reduction in longevity [34] or lowered cognitive performance in adulthood [35]. Any long-term costs associated with compensatory growth in methionine-supplemented nestlings have not been considered in previous studies. We here show that methionine supplementation did not have long-term effects in terms of post-fledgling recruitment rates, although our analysis lacks power due to a low number of recruits. Additional work exploring the long-term effects of methionine-induced changes in growth trajectories, preferably in the laboratory where individuals can be followed throughout their lifetime, would be interesting. Most studies are based on alteration of the growth trajectory through changes in dietary intake [33], but methionine-induced alteration of growth relate to changes in resource allocation made by the individuals. Hence, an explicit comparison of the consequences of these different causes of alteration in growth trajectories could be worthwhile.

Blue tits undergo rapid development in the nest, and reach their final skeletal size 14 days post hatching. Not surprisingly, their growth trajectory is canalized [36]. This strong canalization of growth probably explains why we find that a methionine-induced short-term allocation away from growth is rapidly compensated for and does not affect final size. We find that the main cost of a methionine-induced alteration of the growth trajectory is an increase in mortality risk (this study, also shown by ref. 7 in another population of the same species). Interestingly, other studies in wild passerines did not find [6], or did not analyse survival. Possibly, blue tits are more sensitive than other passerines for changes in growth during ontogeny. The mortality costs are expressed mainly in the smallest nestlings (as indicated by the strong effect of initial size before the experiment on mortality). Because blue tits have larger brood sizes than the other passerines studied, a reduction in growth of the smallest nestlings may be particularly detrimental for its survival as it will be rapidly outcompeted by its larger siblings.

Another explanation for the higher mortality in methionine-supplemented nestlings is that a sudden intake of a relatively high dose of pure methionine has negative effects. However, methionine is known to be toxic at concentrations five times or more above the advised level we used [37]. An increase in mortality has not been reported in great tit nestlings, which are of a similar size as blue tits, where the same weight-based dosage of methionine was used [8]. A high intake of methionine induces an infection-like physiological state where the immune system under influence of pro-inflammatory cytokines monopolises much of the body's resources. In the natural environment, where resources are limited and where competition between siblings is severe (especially in blue tits which have large broods), such effects are likely to have severe consequences, leading to increased mortality, such as we documented here. In terms of experimental supplementation of methionine in wild populations, future studies may wish to include simultaneous food ingestion (with and without methionine) as is done in poultry studies, which may reduce mortality while increasing immunocompetence.

A further possible reason for why we did not find a strong benefit of an increased immunocompetence in the parasite-rich nests probably is the weak influence of fleas on the development of nestling. In this experiment, the presence of fleas reduced the body mass of nestlings on day 9, although this effect disappeared later on in the nestling development (see above). The strongest negative effect of the hen flea ectoparasites was physiologically, revealed by a lowered haematocrit of nestlings, indicating anaemia and a poor nutritional status [38]. Our hen flea manipulation was carefully done in order to stay within the natural range. Because ectoparasites have a strong immunodepressing effect, adding exaggerated numbers to a nest likely has a strong impact that overwhelms all other effect. In blue tits, a reduction in growth (but also in PHA response) becomes apparent when adding 200 fleas [39]. In general, manipulation of ectoparasite load typically either has no effect on PHA-response [22], [40], [41], [42], [43], or lowers PHA-response [39], [44], [45].

### Humoral immune defence and methionine supplementation

In addition to the cellular PHA-response, we quantified the concentration of immunoglobulins in nestlings' blood, as an index of humoral immune defences. Immunoglobulins may also be involved in the defence against ectoparasitic arthropods [46]. We observed a clear tendency (0.05<P<0.10) of methionine supplementation to decrease nestlings' total Ig levels, both in nests with low and high ectoparasite load. This pattern was, however, only marginally significant, most likely due to the fact that we had (because of logistic reasons) relatively low sample sizes for Ig levels. We believe that the different directions of the main effect of methionine-supplementation on the cellular component (which was increased) versus the humoral component (which tended to decrease) of the immune system may indicate a trade-off between these components. Methionine specifically enhances cell-mediated immune responses through a pathway that involves gluthathione, which is known to promote the cell-mediated immune response at the expense of the humoral response [25], [26]. Trade-offs within the immune system have been to date largely unexplored in ecological studies, but are clearly important for understanding the functional aspects of immune defence in the wild [e.g. 5]. Our findings thus indicate an interesting avenue for future experimental work in exploring such trade-offs further.

### Conclusions

There is at present good evidence that methionine supplementation enhances cell mediated immune responses during ontogeny, since all studies on wild birds published to date found higher PHA-response in methionine supplemented nestlings several days after methionine supplementation had stopped. We here find that this increase in PHA-response is context dependent, since it occurs only if the ectoparasite load in the nest is high. A methionine-altered physiology reduces growth and increases mortality in blue tits, but this is a short-term cost expressed only during the period when methionine is supplementary fed, and resources are allocated away from growth, presumably into immune defence. In terms of final size, we here find – contrary to our expectations – no clear benefit of an increased allocation to immune defence in the parasite-rich environment. Additional work on describing the benefits of investment in immune defences is needed, especially emphasizing long-term effects, in order to close the gap between studies focusing on ontogeny and life history.

## Materials and Methods

### Ethics statement

All animals were handled in strict accordance with good animal practice as defined by the relevant national animal welfare bodies (Uppsala province, Sweden).

### Study system

The experiment was conducted during May and June 2004 in a nest-box blue tit population, in the southern part of the Swedish island of Gotland in the Baltic Sea (57° 10′ N, 18° 20′ E). In our population females lay a single clutch per season, consisting on average of 10–12 eggs, which they incubate for approximately two weeks. Young reach their final size usually two weeks post-hatching, and fledge at the age of 18–22 days. Blue tits are commonly hosts of haematophagous hen fleas [47], [48]. Hen fleas are nest-based parasites: adult fleas feed on hosts' blood, but otherwise live in the nest material; larvae feed on detritus from the nest. Hen fleas are detrimental to their hosts, impairing growth and survival of nestlings [e.g. 49].

### Parasite treatment

Before the start of the breeding season, nest-boxes were cleaned and old nests collected to obtain fleas for subsequent infestations. Boxes were regularly monitored to determine laying date of the first egg, clutch size and hatching date. Hatching date was defined as the day when the first chick hatched ( = day 0). One day after hatching nests were assigned to parasite treatment groups and the number of fleas in nests was manipulated. Nests from one group, hereafter parasitized nests (N = 21; one nest was predated between day 9 and 13), received additional 40 adult fleas each, whereas nest material of the second group, hereafter deparasitized nests (N = 21), was microwaved for several minutes to kill all nest-based parasites [49]. To remove immigrating fleas, deparasitized nests were additionally heat-treated two more times during the nestling period. Parasite treatment was altered within the sequence in which nests were available. Parasite treatment groups did not differ in clutch size (t_40_ = 0.91; p = 0.37), hatching date (t_40_ = 0.40; p = 0.69) or number of hatchlings (t_40_ = 0.73; p = 0.47). Shortly before nestlings fledged, nest material from both experimental groups, as well as from a control group of non-manipulated nests, was replaced with a layer of dry moss. Collected nests were frozen, and the number of adult fleas was later counted.

Because only in one group nests were microwaved, potentially the treatments might have also differed in other aspects than flea number, such as humidity or presence of other ectoparasites. Nevertheless, it seems unlikely that these factors affected nestlings' development. Inspection of collected nest material has shown that other ectoparasites in blue tit nests in our population occur sporadically.

### Methionine treatment and growth measurements

On day 3, nestlings were individually marked by clipping a unique combination of their nails, weighed with an electronic balance to the nearest 0.1 g and assigned to methionine treatment (control or supplemented). Methionine treatment was alternated within the weight hierarchy, and the treatment of the heaviest nestling was decided at random. Methionine-treated nestlings received 50 µl of 0.1 g ml^−1^ methionine (Sigma, code M9500) suspension in water on day 3 and 4, and 100 µl on day 5 and 6 [see 7].This way nestlings were supplemented 1–2 mg of methionine per g body mass. This dose corresponds to that applied in poultry [16]. Control nestlings received the same volume of water. On day 6 and 9 nestlings were weighed again. Growth during, and after the period of methionine supplementation, was calculated as body mass gain between day 3 and 6, and between day 6 and 9, respectively. On day 9 nestlings were ringed in order to allow life-long individual identification.

### Physiological measurements

On day 13 nestlings were weighed with a spring balance to the nearest 0.1 g and challenged with PHA (Sigma, code L8754). PHA is a lectin from red kidney bean (*Phaseolus vulgaris*) that has mitogenic properties to many cell types, including T lymphocytes. Injected under the skin PHA produces a swelling response, which is carried out by T-lymphocytes, macrophages, basophils and heterophils [17]. PHA-response involves innate and adaptive components of the immune system, thus, it is a multifaceted index of cutaneous immune activity. We followed a simplified protocol [50], and injected 0.04 ml of 5 mg ml^−1^ PHA solution in saline intradermally, into the right wing web. Prior to PHA injection, to ensure precision and repeatability of the measurement, feathers were removed from the place of injection and the thickness of the wing web was measured to the nearest 0.01 mm with a thickness gauge (Mitutoyo 700-117SU, modified by the removal of a spring). The swelling response was measured 24 h (±1 h) after the injection (two measurements; repeatability: 97.3%; F_375, 376_ = 72.93; p<0.0001). PHA-response is defined as the difference between the mean post-injection thickness and pre-injection thickness of the wing web. One nestling did not show a measurable response (possibly because of a failed injection) and was not included in the analysis. PHA intra-dermal injections and measurement of the response swelling of all nestlings were done by the same persons (injections by JEB and response by NP, respectively).

On day 16 blood samples (max. 100 µl) were collected to heparinized capillaries. Capillaries were sealed and stored in a cool box until they were centrifuged later the same day. Haematocrit (cell fraction in the total sample volume) was measured with a digital calliper, and plasma and blood cells were separated. Plasma was stored at −20°C until immunoglobulin (Ig) analysis.

Nestlings' total Ig concentration was determined with an indirect enzyme linked immunosorbent assay (ELISA) using commercial antichicken IgG antibody (10 µl mL^−1^, Sigma, code C6409). This method has been validated for several wild bird species, including *Parus* species and the blue tit see [refs. 51], [52], [53 for details]. In the Ig analysis, 96-well microplates (ImmunoPlate Maxisorp, Nunc Co., Nunc A/S, Roskilde, Denmark) were first coated overnight at 4°C with IgG antibody. After emptying, the wells were saturated for 1 h with 1% bovine serum albumin (BSA, Roche Diagnostics GmbH, Manheim, Germany) prepared in phosphate-buffered saline (PBS, pH 7.4), and then washed three times with PBS-Tween 20 (0.25%). Samples were diluted with 1% BSA/PBS and each sample incubated in duplicates (50 µl per well, sample dilutions 1∶100 and 1∶2000) for 3 h at room temperature. Pooled plasma samples of nestlings were used as calibrators and they were prepared as serial dilutions for generating the standard curve. Total Ig levels of the samples are presented relative to this standard. Arbitrary value of 10^6^ units equals the mean level of the individuals of the pooled sample. After washing, alkaline phosphatise conjugated antichicken IgG antibody (Sigma, code A9171) was added and the plates were incubated overnight at 4°C (dilution of 1∶10 000). Finally, after last washing, P-nitrophenyl phosphate (1 mg mL^−1^, Sigma Chemical 104 Phosphatase Substrate) in a diethanol amine buffer (1 mol L^−1^, pH 9.8) was applied. The optical density was read at 405 nm with a plate reader (Multiskan Ascent, Therma Oy, Finland).

Because of logistic difficulties the Ig level data are missing for 100 nestlings (48 methionine-treated and 52 control) and haematocrit data are missing for 29 nestlings (17 methionine treated and 12 control).

### Final size

On day 16, i.e. shortly before fledging, we measured the final size of nestlings. Body mass was measured with a spring balance to the nearest 0.1 g, tarsus length with a digital calliper to the nearest 0.1 mm (two measurements were taken, with the exception of one nest; repeatability: 98.3%; F_366, 367_ = 115.14; p<0.0001) and wing length with a ruler to the nearest millimetre. In one nest wing length was not measured.

### Statistical analysis

Growth, morphology and physiological measurements were analysed using linear mixed models, with parasite treatment, methionine treatment and their interaction fitted as fixed effects, and brood nested in parasite treatment fitted as a random effect. PHA-response and Ig levels were log_10_-transformed to normalize distribution. In case of a significant interaction, differences between groups were assessed with Tukey post-hoc test. Nestling mortality and recruitment (binary responses) were analysed using generalized linear mixed models with binomial errors and logit link. Brood was fitted as a random effect and methionine treatment, parasite treatment and their interaction were fitted as fixed effects. Body mass on day 3 was also included as a fixed effect in the analysis of mortality. Fore some individuals (see above) we did not collect the full data-set; therefore sample sizes differ between tests. Analyses were conducted using JMP 5.0 (SAS Institute), except for the mortality and recruitment analyses, which were performed with S plus 6.1 (Insightful Corporation).
